# Vogt–Koyanagi–Harada Syndrome in a Ugandan: Diagnostic and Therapeutic Challenges

**DOI:** 10.1155/2019/5192754

**Published:** 2019-09-16

**Authors:** Felix Bongomin, Francis S. Onen, Mark Kaddumukasa

**Affiliations:** ^1^Department of Internal Medicine, College of Health Sciences, Makerere University, P. O. Box 7072, Kampala, Uganda; ^2^Department of Medical Microbiology & Immunology, Faculty of Medicine, Gulu University, P. O. Box 166, Gulu, Uganda; ^3^Department of Ophthalmology, College of Health Sciences, Makerere University, P. O. Box 7072, Kampala, Uganda

## Abstract

**Background:**

Vogt–Koyanagi–Harada (VKH) syndrome is a multisystemic autoimmune disease of uncertain pathogenesis. Infectious aetiology has been proposed which is suggested to lead to the loss of melanocytes in the skin, inner ear, meninges, and uvea in those who are genetically predisposed. Information regarding VKH syndrome is scanty among the African population.

**Case Presentation:**

We report a 28-year-old HIV-uninfected Ugandan woman who had previously been well and presented with chronic bilateral panuveitis; symmetrical vitiligo patches on the head, trunk, and upper limbs; tinnitus; and poliosis of the scalp hair, eyelashes, and eyebrows. A flu-like syndrome preceded this. Several weeks of prednisolone and azathioprine therapy resulted in remarkable improvement of the ocular and inner ear symptoms.

**Conclusion:**

A high index of suspicion is required in diagnosing VKH syndrome, even in sub-Saharan Africa where the disease is reported to be rare. Initiation of prompt and appropriate treatment prevents blindness and other complications.

## 1. Background

Vogt–Koyanagi–Harada (VKH) syndrome is an established autoimmune multisystem disease that affects pigmented structures in the body such as the eye, inner ear, meninges, and skin [1, 2]. Vogt, Koyanagi, and Harada independently described the initial set of patients at different stages of the disease [2–4]. The classic clinical course of the VKH syndrome can be divided into four stages [2]: the prodromal, acute uveitic, convalescent, and chronic recurrent stages. The prodromal stage precedes the acute uveitic stage by a few days and may mimic a viral infection. The acute uveitic stage lasts several weeks and is followed by the convalescent stage, in which the depigmentation of the tissues is more evident. Some patients enter the chronic recurrent stage with recurrent bouts of anterior uveitis. VKH syndrome is uncommon, affecting mainly darkly pigmented Asian, Middle-Eastern, Hispanic, and Native American populations [5]. The disorder is less common in Caucasians and blacks from sub-Saharan Africa [6]; however, the clinical manifestations are the same as in patients from high prevalence regions [7]. Several cases of VKH syndrome have been reported in North Africa, especially Tunisia where VKH syndrome accounts for about 10 to 15% of uveitis cases [8–10]. To date, only 3 cases of VKH syndrome in Eastern Africa have been reported in the literature [11, 12]. Herein, we report another case of VKH syndrome in Eastern Africa, and the first case of VKH syndrome to be described in a Ugandan.

## 2. Case Presentation

We report a 28-year-old HIV-negative Ugandan woman with no known chronic illness. She presented to the eye clinic at Mulago National Referral Hospital (Kampala, Uganda) with a 2-week history of sudden onset blurring of vision in both eyes, especially in the mornings. This was preceded by a 1-week history of low-grade intermittent fevers, sore throat, running nose, and a nonproductive cough. These symptoms were attributed to a viral upper respiratory tract infection that was managed conservatively. She denied any history of headache, neck stiffness, floaters, excessive tearing, or reddening of the eyes but reported itching of both eyes. There was no history of chronic eye disease, trauma, or previous surgery on the eyes. There was a family history of blindness, which affected one of her four paternal uncles and his son who died under unclear circumstances. Notably, both relatives developed sudden loss of vision and had vitiligo-like skin patches.

During this visit, eye examination revealed a best-corrected visual acuity (BCVA) of 6/36 in both eyes and intraocular pressures (IOP) of 10 mmHg and 11 mmHg in the left and right eye, respectively. On slit lamp examination, pigmented keratic precipitates (KPs), posterior synechiae, and vitreous inflammatory cells were noted. She was managed for an acute uveitis for which she was prescribed Maxitrol® eye drops (active ingredients being dexamethasone, neomycin, and polymyxin B) with minimal improvement after 2 weeks of treatment.

Two weeks later, she developed a sudden onset frontal headache which was throbbing in character and associated with reddening, tearing, and pain involving both eyes. She also reported transient episodes of visual loss but denied any history of neck pain, photophobia, phonophobia, loss of consciousness, or convulsions. During this time, she also noticed impaired hearing in the right ear with bilateral tinnitus, especially in quiet places. There was no associated discharge, dizziness, vertigo, nausea, or vomiting. She was initiated on oral prednisolone 10 mg daily for one week with reported improvement in the symptoms. Her vision improved bilaterally though it remained blurred with floaters. Hearing also improved bilaterally, but the tinnitus persisted.

Five weeks after the onset of the symptoms, she developed an intensely itchy and painful papular rash, which was generalised. This healed after a week but left some hypopigmentation around the face and trunk. There was also associated whitening of her scalp hair, eyebrows, and lashes. However, there was no hair loss/balding.

**Figure 1:**
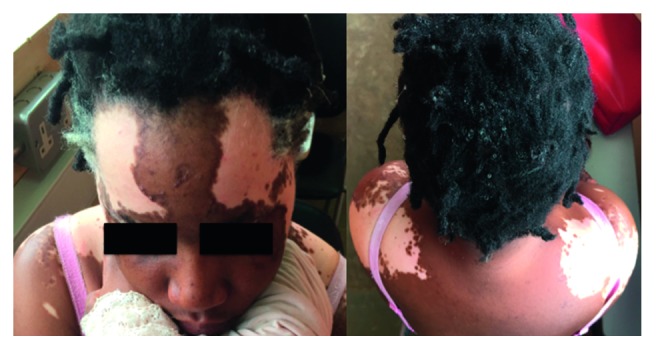
Multiple, symmetrical, “chalk-white”, sharply demarcated, nonscaly patches on both sides of the forehead and upper back, discrete scattered macules on upper and lower limbs, and the torso with intact sensation. Blanching was observed in these patches. White patches of hair involving the scalp hair, eyebrows, and eyelashes. No evidence of hair loss.

On clinical examination, she had multiple, “chalk-white”, sharply demarcated, nonscaly patches on both sides of the forehead and upper back, discrete scattered macules on the upper and lower limbs, and the torso with intact sensation. Blanching was observed in these patches. Patches of white hair involving the scalp hair, eyebrows, and eyelashes were also noted. She had no evidence of hair loss, and her fingernails and toenails were normal (Figure 1). The thyroid gland was normal, and she was clinically euthyroid. Other systemic examination was unremarkable. Her vitals were within normal ranges: temperature = 36.7°C, BP = 120/80 mmHg, PR = 79 bpm, RR = 16 bpm, and SPO_2_ 97% on ambient air.

**Figure 2:**
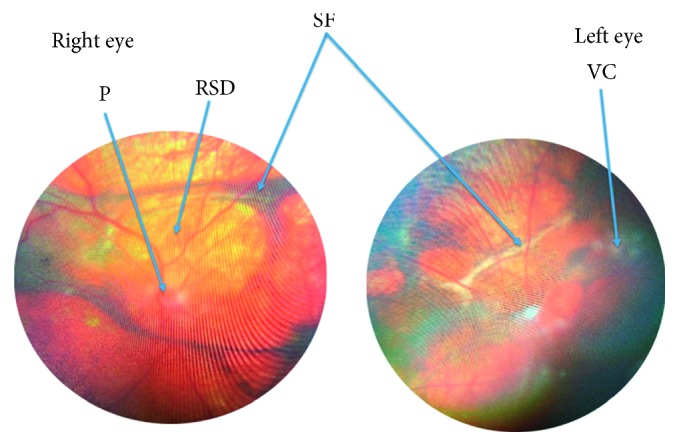
Fundus photograph showing papillitis (P) with blurred optic disc, vitreous cells (VC), and retinal serous detachment (RSD) in the right eye and subretinal fibrosis (SF) in both eyes.

Eight weeks after the onset of her disease, ocular examination revealed a BCVA of 6/36 in both eyes (same as previous), with IOP of 11 mmHg bilaterally. She had normal eyelids, with patches of white eyelashes. Her conjunctivae were normal. Keratic precipitates were noted on the endothelium of the cornea in both eyes. She had posterior synechiae, and the lenses were clear in both eyes. The inflammatory cells and flares were observed in the vitreous. Fundoscopy revealed a “sunset glow fundus”, serous retinal detachment, blurring of the optic disc margin, papillitis, and normal retinal vessels bilaterally (Figure 2). However, inflammatory cells (“snow balls”) were observed in the vitreous of the left eye.

A diagnosis of VKH syndrome was considered, as possibly a complete VKH syndrome in the chronic stage. Full blood count, erythrocyte sedimentation rate, antinuclear antibody tests, and chest radiograph were normal. The Mantoux test and a venereal disease research laboratory test were all negative. Oral prednisolone was restarted at a dose of 15 mg daily for two weeks in addition to oral azathioprine at a dose of 50 mg daily for one month and possible refills for 6 months. She received counselling regarding her disease and on the irreversibility of her vitiligo and poliosis, which were of concern to her. She continues to attend follow-up clinic visits.

## 3. Discussion

To the best of the authors' knowledge, the abovementioned case represents the first case of VKH syndrome to be reported from Uganda. VKH disease is reported to be rare among black Africans. Timely diagnosis and initiation of the appropriate treatment is vital so as to delay complications, and this may be difficult to achieve in settings such as Uganda. Studies have shown that females are more commonly affected [2] such as our index case who was a young Ugandan woman with the disease. Our patient presented initially with a nonspecific febrile illness (probably viral) that preceded the development of the ocular, auditory, and integument manifestations. She never reported any of the known neurological manifestations of the disease such as meningismus or neck stiffness. It may well be that this is not a common presentation in African settings.

The diagnosis of VKH syndrome is clinical and is based on the following criteria: (1) absence of a previous history of ocular trauma or surgery; (2) no evidence of other ocular diseases; (3) early bilateral ocular involvement (with focal areas of subretinal fluid or serous retinal detachment) or late bilateral ocular involvement (depigmentation, sunset glow fundus, Dalen-Fuchs nodules, and migration or accumulation of the pigmented epithelium of the retina); (4) history or presentation of auditory and/or neurological symptoms; and (5) cutaneous symptoms that appear during or after the neurological and ocular manifestations. A patient who meets all of the five criteria is classified as having a complete VKH syndrome. Incomplete VKH syndrome is diagnosed when only criteria 1, 2, and 3 plus 4 or 5 are met, and probable VKH disease when only criteria 1, 2, and 3 are met [13]. Our patient had a complete VKH syndrome, meeting all 5 of the abovementioned criteria. Serous retinal detachment and optic disc swelling, which are distinctly uncommon occurrences during the chronic phase of VKH syndrome, were observed in this patient.

Early diagnosis with prompt and adequate treatment with corticosteroids and other immunosuppressive agents (such as methotrexate, etanercept, tacrolimus, cyclosporine, mycophenolate mofetil, azathioprine, cyclophosphamide, chlorambucil, and adalimumab) may halt disease progression and prevent recurrences and vision loss [14]. Corticosteroids are the preferred first-line therapy and should be administered for at least 6–12 months. Intravenous immunoglobulin and rituximab are reserved for steroid resistant disease [2, 15].

Prognosis depends greatly on how early the disease is diagnosed and correctly treated [14]. Auditory symptoms respond well to the treatment and are generally completely reversed in 2 to 3 months. However, cutaneous lesions are permanent. Ocular symptoms have extremely variable prognosis although generally favorable with younger patients suffering a lower rate of ocular complications [2]. Our patient had subretinal fibrosis, which indicates that she was not adequately treated initially, hence the ongoing impaired visual acuity [16, 17].

The key limitations in the management of the present case lie in the incompleteness of the ophthalmological investigations and lack of formal audiometric tests. It is likely that the immunosuppressive agents we used were given at a suboptimal dose. However, the patient reported a significant improvement in both ocular and auricular symptoms.

In conclusion, although VKH syndrome is rare in sub-Saharan Africa, the disease should be suspected in patients with no previous eye trauma and in those who present with bilateral panuveitis along with other symptoms consistent with the diagnosis of VKH syndrome. Diagnostic and therapeutic challenges are common in resource-limited settings like Uganda, partly due to physicians' lack of experience in the management of this rare syndrome but also because of issues related to affordability and access to essential diagnostic tools and immunosuppressive agents. However, early diagnosis and appropriate treatment of VKH syndrome prevent the onset of the irreversible integumentary symptoms as well as the sight-threatening complications of the disease.
